# Heart Rate Variability as a Digital Biomarker in Adolescents and Young Adults Receiving Hematopoietic Cell Transplantation

**DOI:** 10.1002/cam4.70609

**Published:** 2025-02-21

**Authors:** Mallory R. Taylor, Miranda C. Bradford, Chuan Zhou, Kaitlyn M. Fladeboe, Jorie F. Wittig, K. Scott Baker, Joyce P. Yi‐Frazier, Abby R. Rosenberg

**Affiliations:** ^1^ Department of Pediatrics University of Washington School of Medicine Seattle Washington USA; ^2^ Ben Towne Center for Childhood Cancer and Blood Disorders Research Seattle Children's Research Institute Seattle Washington USA; ^3^ Biostatistics Epidemiology and Analytics in Research Core Seattle Children's Research Institute Seattle Washington USA; ^4^ Center for Child Health, Behavior, and Development Seattle Children's Research Institute Seattle Washington USA; ^5^ University of Washington School of Medicine Seattle Washington USA; ^6^ Clinical Research Division Fred Hutchinson Cancer Research Center Seattle Washington USA; ^7^ Department of Psychosocial Oncology and Palliative Care Dana‐Farber Cancer Institute Boston Massachusetts USA; ^8^ Department of Pediatrics Boston Children's Hospital Boston Massachusetts USA; ^9^ Department of Pediatrics Harvard Medical School Boston Massachusetts USA

**Keywords:** adolescents and young adults, digital biomarkers, heart rate variability, hematopoietic cell transplantation, psychosocial

## Abstract

**Introduction:**

Adolescents and young adults (AYAs) receiving hematopoietic cell transplantation (HCT) are at high risk for poor psychosocial outcomes. Heart rate variability (HRV), a surrogate for autonomic nervous system activity, is a promising digital biomarker that has been linked to important outcomes. The objectives of this study were to prospectively describe the trajectory of HRV among AYAs receiving HCT and explore the association between HRV and patient‐reported outcomes (PROs).

**Methods:**

This was a multi‐site study embedded in a randomized trial among AYAs receiving HCT (NCT03640325). We collected sequential 24‐h HRV metrics, including the standard deviation of normal‐to‐normal beats (SDNN), root‐mean‐square of successive differences (RMSSD), as well as frequency domain measures. PRO surveys queried anxiety, depression, quality of life, hope, and resilience at baseline and 3 months. We summarized outcomes using descriptive statistics, and Pearson correlation coefficients were used to examine the relationship between HRV and PROs.

**Results:**

Thirty‐nine HRV recordings were collected from *n* = 16 participants aged 12–21 years. There was a moderately strong correlation between inferior baseline HRV and higher anxiety and depression (anxiety: *r* = −0.35 (*p* = 0.18) for SDNN, *r* = −0.47 (*p* = 0.07) for RMSSD; depression: *r* = −0.26 (*p* = 0.34) for SDNN, *r* = −0.39 (*p* = 0.14) for RMSSD). Among participants with elevated baseline anxiety, higher HRV suggested greater improvement in anxiety over time (*r* = −0.66 (*p* = 0.08) for SDNN, *r* = −0.31 (*p* = 0.45) for RMSSD).

**Conclusions:**

There was a correlation between HRV and PROs in this study, and among those with elevated anxiety, HRV predicted improvement over time. Digital biomarkers may augment behavioral intervention design and implementation.

## Graphical abstract

Heart rate variability was associated with anxiety and depressive symptoms among AYAs receiving hematopoietic cell transplantation. Digital biomarkers of psychosocial symptoms may augment behavioral intervention design and implementation.

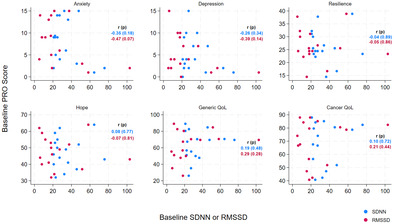

## 1 Introduction

Adolescents and young adults (AYAs) with cancer are at high risk for poor psychosocial outcomes. In addition to the more visible physical side effects of cancer and its treatment, AYAs endorse significantly worse mental health than their non‐cancer peers [1]. Cancer disrupts the normal trajectories of identity development, educational and vocational attainment, and emerging independence that are hallmarks of adolescence and early adulthood.

These challenges may be more pronounced for AYAs receiving hematopoietic cell transplantation (HCT) to treat their disease. HCT is a highly intensive treatment with increased risks of short‐ and long‐term morbidity. Nearly half of pediatric patients experience significant increases in anxiety, depression, and loneliness following transplant, and over 80% of patients report moderate post‐traumatic stress symptoms 3 months post‐HCT [2]. Emerging experimental and translational data are beginning to shed light on the complex biopsychosocial mechanisms relevant to cancer [3]. However, the overwhelming majority of this research is focused on adults with cancer, leaving a gap in our understanding of the biobehavioral landscape in AYA oncology.

Identifying biomarkers of psychosocial constructs is a key step toward understanding the converging psychological and biological processes in cancer. Fortunately, new tools in behavioral quantification and digital health technology (DHT) can facilitate scientific progress toward this goal [4]. Digital biomarkers of psychosocial states, such as stress, symptom burden, and mood, can improve care delivery and patient outcomes [5, 6]. One such digital biomarker is heart rate variability (HRV), which is the fluctuation in intervals between successive heartbeats. HRV is a multidimensional measure of autonomic nervous system regulatory activity and has been associated with meaningful clinical and psychosocial outcomes [7]. Indeed, lower HRV (indicating less ‘autonomic flexibility’) has been associated with anxiety [8], depression [9], and adolescent emotion regulation [10], as well as cancer‐related fatigue and mortality [11, 12]. Impaired HRV has been observed in the adult HCT and HCT survivor population [13, 14], and there have been few studies in AYAs receiving transplant.

The objectives of this study were to prospectively describe the trajectory of HRV among AYAs undergoing HCT and explore the relationship between HRV and patient‐reported outcomes (PROs). We hypothesized that HRV would correlate with PROs, and that baseline HRV would predict change in PROs over time.

## 2 Materials and Methods

### 2.1 Design

This was a prospective study embedded in a randomized clinical trial (RCT) testing the Promoting Resilience in Stress Management (PRISM) resilience intervention compared to usual psychosocial care in AYAs with cancer receiving HCT (NCT03640325) [15]. The primary aim of the trial was to determine the intervention's effects on patient‐reported anxiety and depression symptoms. Participants for this optional ancillary HRV study were recruited from 3 sites (Seattle Children's Hospital, St. Jude Children's Research Hospital, and Children's Hospital of Los Angeles) from January 2019 to March 2023. The study was approved at each hospital's Institutional Review Board.

### 2.2 Participants

Eligible participants were those aged 12–24 years within 4 weeks of receiving an allogeneic or autologous HCT for a malignancy or cancer predisposition syndrome who spoke English or Spanish and were cognitively able to participate in study activities. Written informed consent was obtained for participants > 18 years old; written informed assent and caregiver consent was obtained for participants 12–17 years.

### 2.3 Study Procedures

Among enrolled participants, we recorded HRV at baseline, 1‐, and 3‐months post HCT to align with RCT time points and salient clinical milestones (stem cell engraftment and transition to less intensive HCT care). PROs were collected at baseline and 3 months and included validated surveys of anxiety and depression (Hospital Anxiety and Depression Scale (HADS)) [16], quality of life (Pediatric Quality of Life (PedsQL) Generic and Cancer Module Teen Reports) [17], hope (Snyder Hope Scale) [18], and resilience [Connor‐Davidson Resilience Scale (CD‐RISC)] [19].

HRV was quantified using the Actiheart 5 wearable device (CamnTech Inc., UKFDA class 2, 510(k) number K052489). The Actiheart 5 is an FDA‐approved, lightweight, wireless electrocardiogram (ECG) monitor with established validity and reliability [20]. The device was attached to a patient's torso via two standard ECG electrodes and worn for a 24‐h period, the gold standard for HRV assessment. Comprehensive HRV output metrics were computed by the Actiheart Software (CamnTech Inc) using a 256 Hz sampling rate, bandwidth of 0.05–55 Hz, and 5‐min epochs. Time and frequency domain measures were derived per established guidelines, including the standard deviation of normal‐to‐normal beats (SDNN), root mean square of successive differences (RMSSD), high frequency (HF), low frequency (LF), and the LF/HF ratio [21].

Prior to analysis, two study team members manually inspected raw ECG data and removed artifacts not detected by the Actiheart software. We identified analytic windows with the longest contiguous segment of high‐quality data and without relevant abnormalities (ectopy, wide QRS intervals, arrythmias). Overnight tracings were preferentially selected to minimize motion artifact and circadian variation. Medical record abstraction identified clinical variables relevant to HRV, including coexisting cardiac conditions, cardioactive medications, abnormal echocardiograms or ECGs, and concomitant infectious or febrile illness. HRV data were collected and analyzed in accordance with the Guidelines for Reporting Articles on Psychiatry and Heart rate variability [22].

### 2.4 Statistical Design and Analysis

We described patient baseline characteristics using counts and percentages or medians and interquartile ranges. We summarized HRV measures by (1) sex and (2) intervention arm at each time point using means, medians, and interquartile ranges. Patient‐reported outcome scores were summarized at each time point using means and standard deviations. Pearson correlation coefficients were used to assess the relationship between baseline HRV and PROs, as well as the relationship between HRV and change in anxiety symptoms from baseline to 3 months in the pooled cohort. There was insufficient 3‐month HRV data in this cohort to reliably perform correlation testing, and so this was not included in this analysis.

## 3 Results

A total of 39 HRV recordings were collected from *n* = 16 patients at the three institutions; eight were randomized to the PRISM intervention, and eight received usual psychosocial care. Participants were aged 12–21 years, 53% were male, and just over half (56%) had a diagnosis of acute myelogenous leukemia (Table 1). All participants received an allogeneic transplant, with cord blood as the most common graft source (47%). Nine participants had ECGs labeled as abnormal within 4 weeks of baseline HRV recordings, but the majority (6/9) were normal pediatric ECG variants, including nonspecific T wave abnormalities (*n* = 3), sinus arrhythmia (*n* = 2), and low voltage QRS (*n* = 1). There were no documented concurrent infections or fevers during the HRV recordings.

**TABLE 1:** Participant characteristics.

Characteristic	(*N* = 16)
Age, median (IQR)	15 (13, 18)
Sex at birth, *n* (%)	
Male	9 (56)
Female	7 (44)
Race/Ethnicity, *n* (%)	
Hispanic	3 (19)
Non‐Hispanic White	12 (75)
Non‐Hispanic Asian	1 (6)
Diagnosis, *n* (%)	
Acute lymphoblastic leukemia	7 (44)
Acute myelogenous leukemia	9 (56)
HCT category^a^, *n* (%)	
Allogeneic	15 (100)
Graft type^a^, *n* (%)	
Bone marrow	3 (20)
Peripheral blood	5 (33)
Cord blood	7 (47)
Intervention arm, *n* (%)	
PRISM	8 (50)
Usual care control	8 (50)
Baseline PRO Scores, mean (SD)	
HADS‐total	13 (9)
HADS‐depression	5 (4)
HADS‐anxiety	8 (5)
Snyder Hope Scale	48 (10)
CD‐RISC	26 (7)
PedsQL cancer	70 (17)
Baseline HRV by sex, median (IQR)	
Female	
SDNN	27 (22–36)
RMSSD	18 (8–27)
HF	100 (17–383)
LF	119 (83–337)
LF/HF	1 (1–5)
Male	
SDNN	27 (25–46)
RMSSD	18 (12–52)
HF	146 (45–571)
LF	154 (131–506)
HF/LF	2 (1–3)
Baseline clinical cardiac data	
Beta blockers, *n* (%)	0 (0)
Cardiac remodeling agents, *n* (%)	3 (19)
Anti‐hypertensive agents, *n* (%)	2 (12)
Abnormal ECG, *n* (%)	9 (60)
Abnormal echocardiogram, *n* (%)	7 (47)
Febrile during reading, *n* (%)	
No	11 (100)
Documented infection during reading, *n* (%)	
No	11 (100)

Abbreviations: CD‐RISC=Connor‐Davidson Resilience Scale; ECG = electrocardiogram; HADS = Hospital Anxiety and Depression Scale; HCT = hematopoietic cell transplantation; HRV = heart rate variability; HF = high frequency; LF = low frequency; PedsQL = Pediatric Quality of Life Inventory; PRISM = Promoting Resilience in Stress Management; PRO = patient reported outcome; RMSSD = root mean square of successive differences; SDNN = standard deviation of normal‐to‐normal beats.

^a^ One participant did not proceed with HCT following partial conditioning due to infection.

Median (IQR) baseline SDNN and RMSSD were 27 (21, 38) and 11 (8, 22) for males and 25 (22, 27) and 13 (4, 19) for females, respectively (Table 1). For reference, published normative adolescent SDNN and RMSSD median (IQR) values are 63 (48, 85) and 59 (45, 88) for males and 66 (46, 87) and 69 (49, 100) for females, respectively (Figure S1) [23]. Among patients with longitudinal data, there were no detectable differences in HRV based on the intervention, nor did we identify consistent patterns for change in HRV metrics over time (Figures S2 and S3).

**FIGURE 1:**
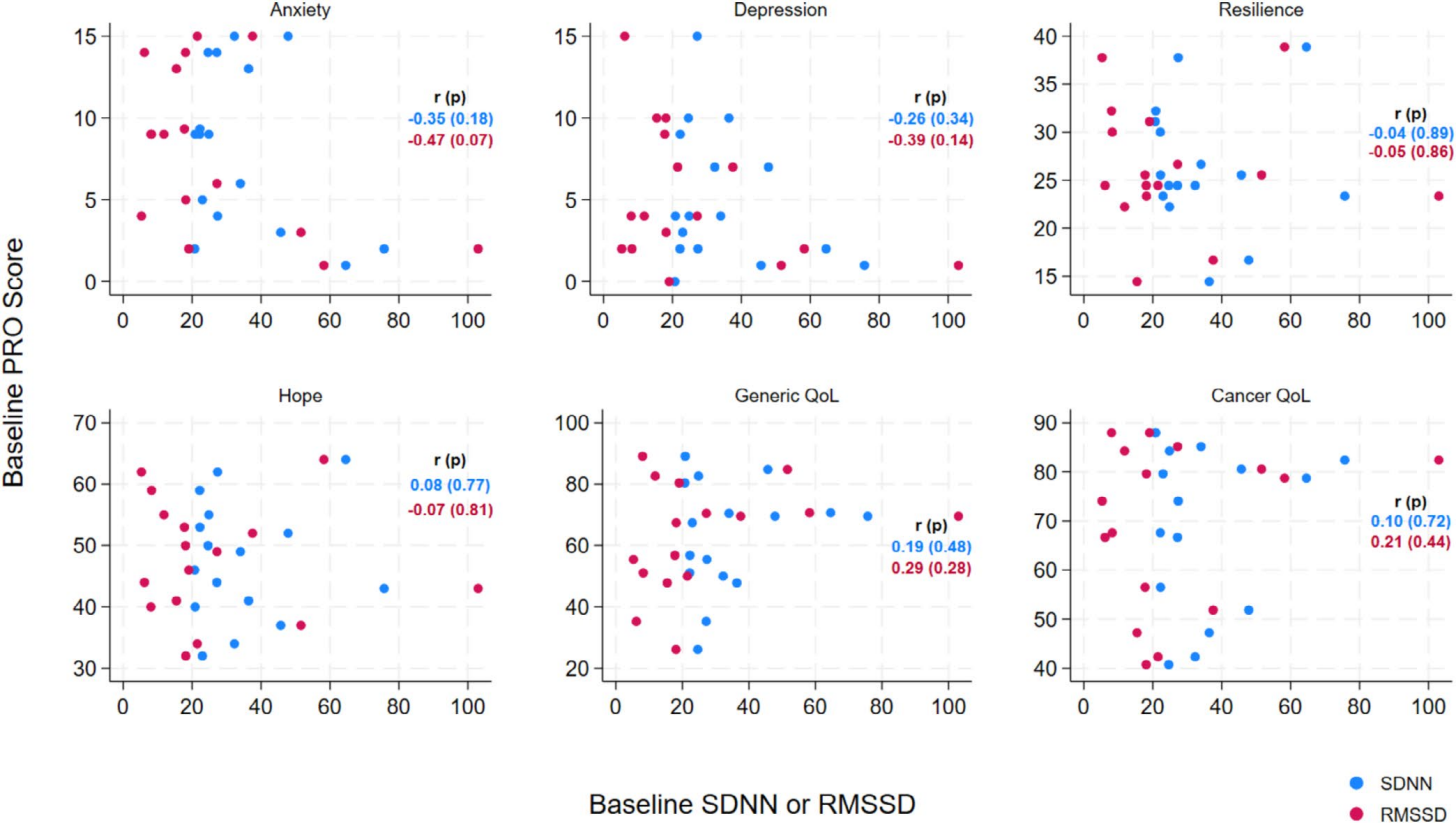
Correlation of baseline HRV with baseline patient‐reported outcomes. *p*‐values indicate probability that the correlation strength occurs by chance.

When examining the relationship between baseline HRV and patient‐reported outcomes, there was a moderate inverse correlation between HRV and anxiety and depression as expected. Participants with lower (inferior) baseline SDNN or RMSSD reported higher anxiety and depression symptoms at baseline (anxiety: *r* = −0.35 (*p* = 0.18) for SDNN, *r* = −0.47 (*p* = 0.07) for RMSSD; depression: *r* = −0.26 (*p* = 0.34) for SDNN, *r* = −0.39 (*p* = 0.14) for RMSSD), (Figure 1). We did not detect meaningful correlations between baseline HRV and other PROs (Figure 1).

**FIGURE 2:**
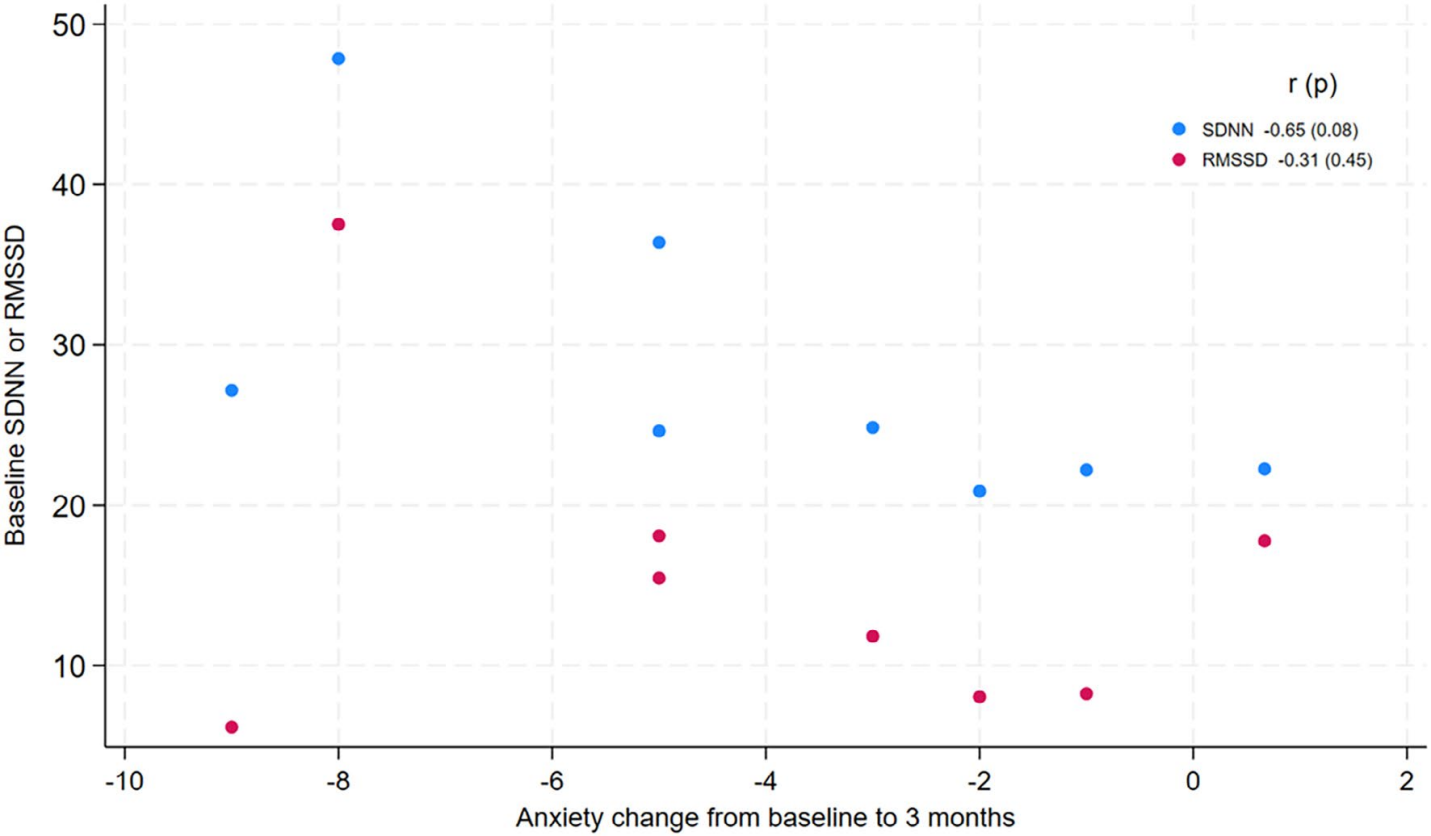
Change in anxiety symptoms over time among participants with elevated baseline anxiety.

Among a subset of patients with baseline anxiety symptoms elevated above the sample median (HADS Anxiety score > 5, *n* = 8), higher baseline HRV correlated with greater improvement in anxiety scores from baseline to 3 months (*r* = −0.65 (*p* = 0.08) for SDNN *r* = −0.31 (*p* = 0.45) for RMSSD), (Figure 2).

## 4 Discussion

In this prospective study of AYAs receiving HCT, there was a moderately strong correlation between measures of HRV and patient‐reported symptoms of anxiety and depression. This relationship was most pronounced among AYAs with elevated symptoms of anxiety, which is consistent with other studies linking reduced HRV and anxiety disorders [8, 24]. We did not detect correlations between HRV and patient‐reported resilience, hope, or health related quality of life. Compared to published values for healthy adolescents, AYA participants had inferior baseline measures of HRV, and this did not improve substantially over time.

Although our numbers are small, these findings are consistent with prior work in this field [8, 9, 25]. One shared pathway that may at least in part explain the relationship between psychological symptoms, HRV, and health impacts involves the autonomic nervous system. Excessive or prolonged sympathetic nervous system activation, which commonly occur with anxiety or worry [8], is a known risk factor for cardiovascular and other chronic diseases [26, 27] and may have important cancer‐related implications as well. Sympathetic nervous system regulation governs aspects of stem cell biology and trafficking, the tumor microenvironment, metastatic potential, oncogene transcription, and immune cell function [28, 29, 30]. Thus, sympathetic nervous system dysregulation has the potential to directly influence cancer biology and clinical outcomes. Leveraging HRV to more fully understand this pathway could be especially useful among AYAs with cancer, given the high prevalence of anxiety and generally inferior cancer‐related outcomes in this population.

Among participants with elevated baseline anxiety scores, more favorable baseline HRV profiles correlated with greater improvement in anxiety scores over time. This may suggest that higher ‘autonomic flexibility’ as indexed by higher HRV identifies a subset of individuals with a greater capacity for responsiveness to an intervention. In this way, digital biomarkers like HRV could augment standard psychosocial or clinical characteristics when interpreting results of behavioral intervention trials and ultimately enhance precision when risk‐stratifying and risk‐adapting resource‐limited interventions. For example, a ‘stepped care’ model that integrates patient‐reported and HRV biomarker data could be used to designate psychosocial risk groups. Higher intensity behavioral intervention (earlier, more frequent, one‐on‐one, specialized, etc.) could then be allocated to higher risk subgroups.

This study had notable limitations that should be considered when interpreting the results. First, although this was a multisite study, we enrolled participants during the peak of the COVID‐19 pandemic, resulting in a small sample size. However, HRV data quality for each participant was excellent, optimized through the use of 24‐h ECG‐derived recordings and stringent quality control procedures. Additional participant characteristics, including prior total anthracycline exposure, physical fitness, or sleep quality, were incompletely captured and could be contributing to HRV metrics. Although this study was embedded in an RCT testing the PRISM behavioral intervention, there were too few longitudinal data points to formally test the effects of the intervention on HRV change over time. With fewer pandemic‐related research restrictions, ongoing work is focused on prospective data collection in a larger patient population.

## 5 Conclusion

In summary, our data support a relationship between HRV and psychosocial symptoms among AYAs receiving HCT. The field of digital psychosocial biomarker science is growing rapidly and provides novel tools to help shape the future of cancer research and clinical care [5]. Wearables that capture HRV offer a minimally burdensome, non‐invasive way to collect real‐time, high‐resolution biometric data that can be integrated into prognostic or therapeutic studies and clinical care. The ‘digital native’ AYA population may be especially suited to the integration of wearable technology into their daily lives, making HRV an appealing biomarker in this group. Additional work should build on this data to validate clinically meaningful HRV cut points in the AYA population. Future studies should also incorporate complementary psychosocial biomarker platforms, such as smartphone digital phenotyping [31] or social genomics [32], that can help facilitate a deeper understanding of complex biobehavioral processes and enhance the infrastructure of precision supportive care in oncology.

## Author Contributions

**Mallory R. Taylor:** conceptualization (lead), data curation (lead), formal analysis (supporting), funding acquisition (lead), investigation (lead), methodology (equal), writing – original draft (lead). **Miranda C. Bradford:** conceptualization (supporting), formal analysis (lead), methodology (supporting), visualization (lead), writing – review and editing (equal). **Chuan Zhou:** methodology (supporting), validation (supporting). **Kaitlyn M. Fladeboe:** methodology (supporting), project administration (supporting), writing – review and editing (equal). **Jorie F. Wittig:** data curation (supporting), project administration (supporting), writing – review and editing (supporting). **K. Scott Baker:** conceptualization (supporting), methodology (supporting), supervision (equal), writing – review and editing (equal). **Joyce P. Yi‐Frazier:** conceptualization (supporting), investigation (supporting), methodology (supporting), supervision (supporting), writing – review and editing (equal). **Abby R. Rosenberg:** conceptualization (supporting), funding acquisition (supporting), methodology (supporting), resources (supporting), supervision (lead), writing – review and editing (equal).

## Ethics Statement

This study was approved by the Institutional Review Board at each participating site.

## Conflicts of Interest

The authors declare no conflicts of interest.

## Supporting information


**Figure S1:** Baseline cohort heart rate variability metrics by sex compared to healthy adolescents. HRV = heart rate variability, SDNN = standard deviation of normal‐to‐normal beats, RMSSD = root mean square of successive differences.

**Figure S2:** Individual (circles) and group mean (lines) heart rate variability metrics by sex over time. HRV = heart rate variability, SDNN = standard deviation of normal‐to‐normal beats, RMSSD = root mean square of successive differences, HF = high frequency, LF = low frequency, T2 = 1 month, T3 = 3 months, T4 = 6 months.

**Figure S3:** Individual (circles) and group mean (lines) heart rate variability metrics by intervention arm over time. HRV = heart rate variability, SDNN = standard deviation of normal‐to‐normal beats, RMSSD = root mean square of successive differences, HF = high frequency, LF = low frequency, T2 = 1 month, T3 = 3 months, T4 = 6 months, PRISM = Promoting Resilience in Stress Management.

## Data Availability

The data that support the findings of this study are available from the corresponding author upon reasonable request.
