# Supplementary figures and images for: Heart Rate Variability as a Digital Biomarker in Adolescents and Young Adults Receiving Hematopoietic Cell Transplantation
Source: Cancer Med. 2025 Feb 21;14(4):e70609. doi: 10.1002/cam4.70609 (PMC11843223; doi:10.1002/cam4.70609)

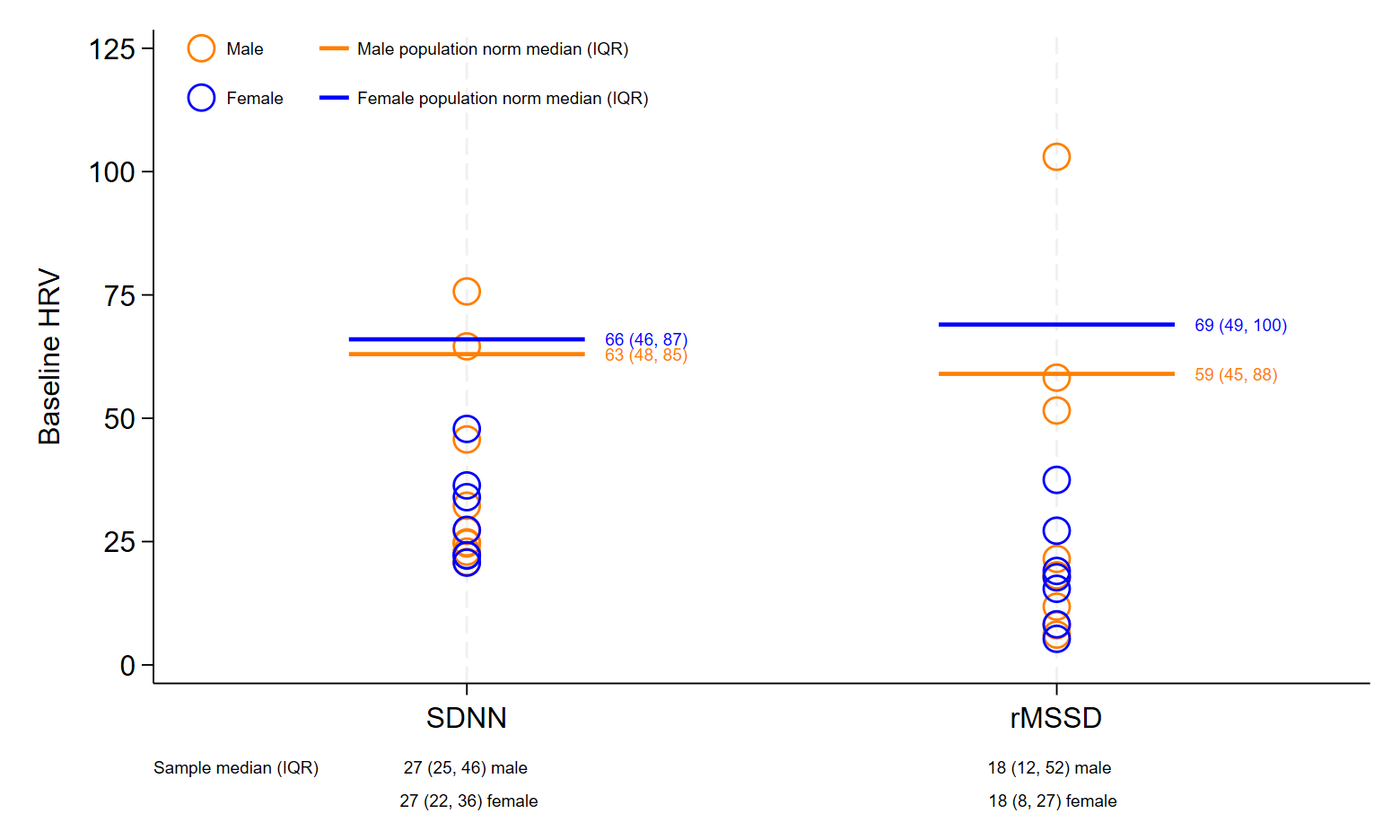

Supplement: Supplementary file 1 — Figure S1: Baseline cohort heart rate variability metrics by sex compared to healthy adolescents. HRV = heart rate variability, SDNN = standard deviation of normal‐to‐normal beats, RMSSD = root mean square of successive differences. [file CAM4-14-e70609-s002.png]

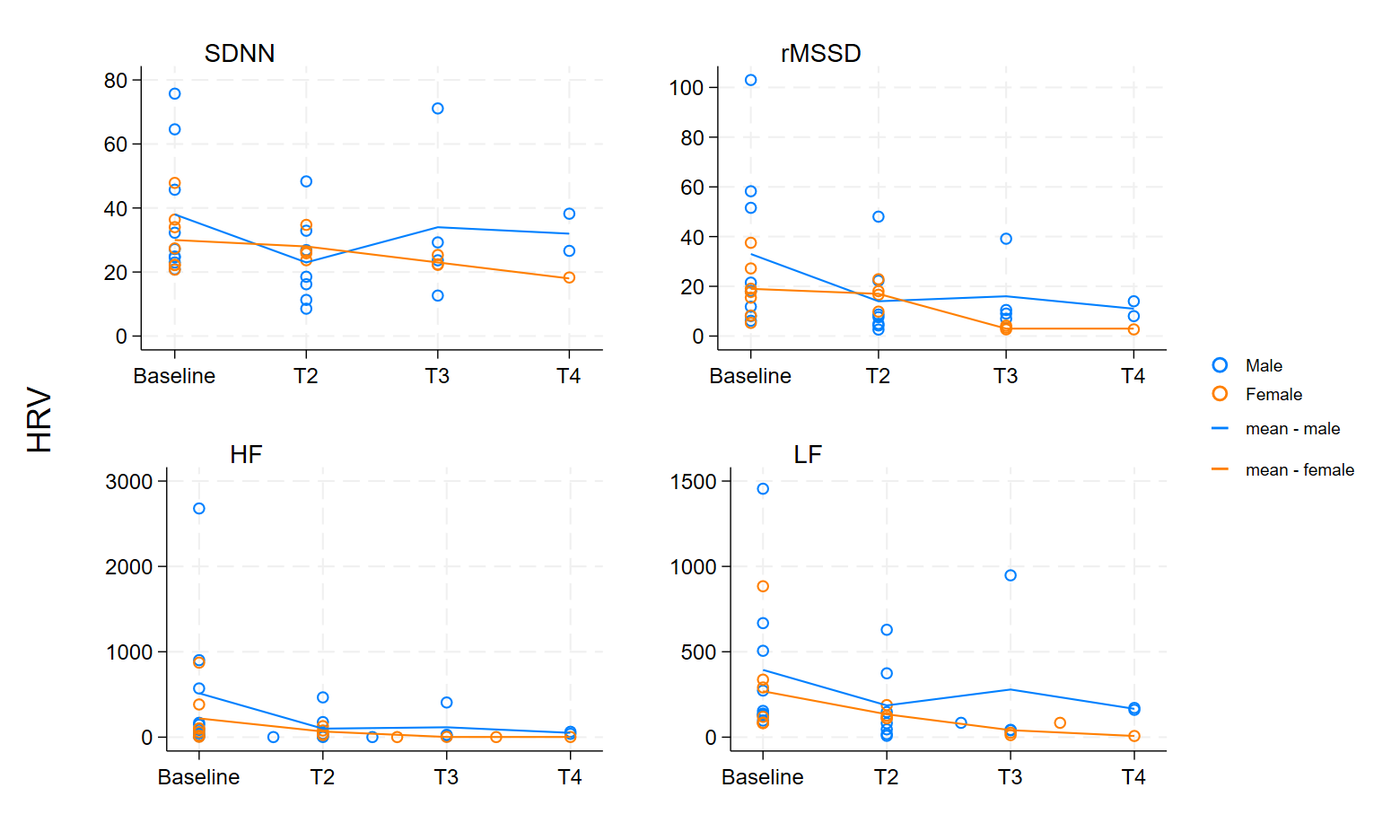

Supplement: Supplementary file 2 — Figure S2: Individual (circles) and group mean (lines) heart rate variability metrics by sex over time. HRV = heart rate variability, SDNN = standard deviation of normal‐to‐normal beats, RMSSD = root mean square of successive differences, HF = high frequency, LF = low frequency, T2 = 1 month, T3 = 3 months, T4 = 6 months. [file CAM4-14-e70609-s003.png]

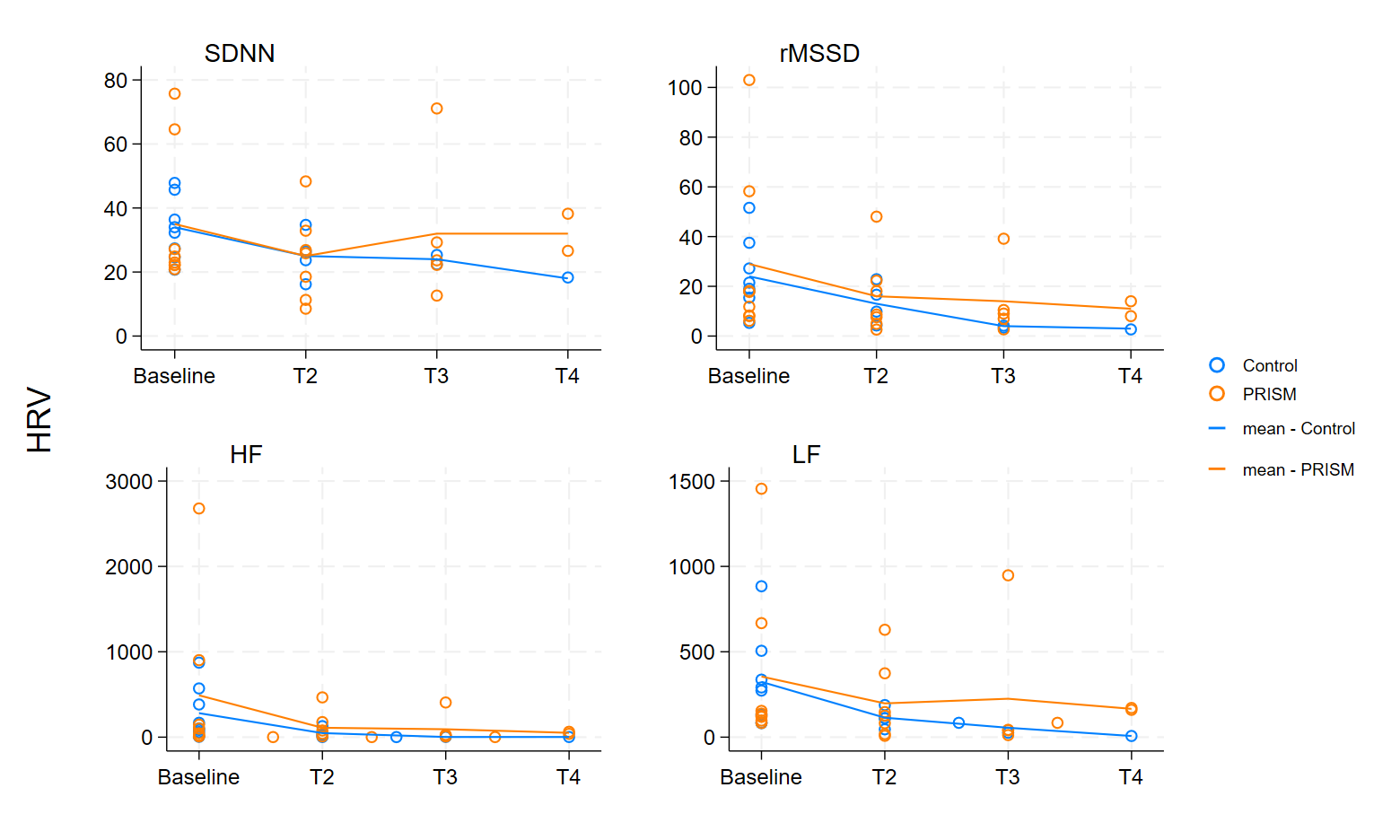

Supplement: Supplementary file 3 — Figure S3: Individual (circles) and group mean (lines) heart rate variability metrics by intervention arm over time. HRV = heart rate variability, SDNN = standard deviation of normal‐to‐normal beats, RMSSD = root mean square of successive differences, HF = high frequency, LF = low frequency, T2 = 1 month, T3 = 3 months, T4 = 6 months, PRISM = Promoting Resilience in Stress Management. [file CAM4-14-e70609-s001.png]
